# A Primrose Path? Moderating Effects of Age and Gender in the Association between Green Space and Mental Health

**DOI:** 10.3390/ijerph13050492

**Published:** 2016-05-11

**Authors:** Elisabeth H. Bos, Leon van der Meulen, Marieke Wichers, Bertus F. Jeronimus

**Affiliations:** 1 Interdisciplinary Center Psychopathology and Emotion Regulation, University Medical Center Groningen, University of Groningen, Groningen 9700 RB, The Netherlands; m.c.wichers@umcg.nl (M.W.); b.f.jeronimus@umcg.nl (B.F.J.); 2 Geoservice, Centre for Information Technology, University of Groningen, Groningen 9747 AJ, The Netherlands; leon.van.der.meulen@rug.nl

**Keywords:** green environment, depression, anxiety, stress, quality of life

## Abstract

This paper explored whether the association between green space and mental health is moderated by age and gender. Questionnaires on psychopathology and quality of life were filled out by 4924 individuals from the general Dutch population and regressed on greenness levels. Green space was associated with better mental health, but only in specific age and gender groups, and only in a 3 km, not a 1 km buffer. The moderating effects of age and gender may be explained by whether or not people have the opportunity to make use of their green living environment.

## 1. Introduction

Over the 20th century, the number of people living in urban areas grew very rapidly, which implies that opportunities for human contact with nature decreased. This is thought to have health consequences because green environments have been associated with both physical and mental health [1,2,3,4]. A number of mechanisms are supposed to underlie the beneficial effect of green space, including air quality, physical activity, social cohesion, and stress reduction [5,6]. 

Regarding mental health, the evidence of the beneficial effects of green space is accumulating [3,7], but strong conclusions are hampered by the scarcity of good-quality studies and the heterogeneity in the definition of green space and exposure assessment. Moreover, some studies suggest that the beneficial effect of green space may be confined to certain age groups [8,9,10], and that also gender may be a moderating factor [10,11]. Such moderating effects of age and gender, however, have been studied only occasionally, and rarely within one and the same study.

In the present study we investigated the association between green space and mental health, and the moderating effects of age and gender in this association. In line with previous studies done in the Netherlands [8,9,12,13] we investigated the effect of green space within a 1 and 3 km radius around the respondents’ home in a general population sample. Expanding on the above studies, we investigated both gender and age interaction effects, and used continuous instead of dichotomous outcomes. Further, we did not only focus on psychopathology but also examined quality of life in order to include positive aspects of health [14]. We expect positive effects of green space, although we expect the size of the effects to differ by gender and age group.

## 2. Methods

### 2.1. Participants

Participants from the general Dutch population were recruited by means of a nation-wide internet study called HowNutsAreTheDutch (in Dutch: HoeGekIsNL). This website was launched by our research group (Interdisciplinary Center Psychopathology and Emotion regulation) on 19 December 2013, with the aim to collect data on mental health using a crowdsourcing procedure. Via several media outlets, individuals were invited to visit the website www.HowNutsAreTheDutch.com and to fill out validated questionnaires on several domains of mental health. Inclusion criteria were adulthood (age ≥ 18) and informed consent on use of the data for scientific research. The study was conducted in accordance with the Declaration of Helsinki and the protocol was approved by the Medical Ethical Committee of the University Medical Center Groningen, The Netherlands (number M13.147422). Details on this study are provided elsewhere [15]. In the first year (2014), 12,503 individuals participated from all around the country. For the present study we selected those participants who filled out the questionnaires relevant to this study. This resulted in a sample of 4924 participants. Mean age of the sample was 47 years (standard deviation (s.d.) = 14, range 18–87) and 65% of the participants were female.

### 2.2. Measures

Psychopathology was assessed with the Depression Anxiety Stress Scale (DASS) [16], which assesses three common domains of psychological symptoms, *viz.* depression, anxiety, and stress, and is known to be sensitive to subthreshold symptoms. The questionnaire consists of 42 self-report items (14 per subscale) rated on a 4-point Likert scale, and covers the past week. The items were summed into a total score (DASS Total). Internal consistency for this total score was high (Cronbach’s α = 0.97).

Quality of life was assessed with the Manchester Short Assessment of Quality of Life (MANSA) [17]. The MANSA measures satisfaction with life as a whole and across a number of different life domains (e.g., work, finance, health, social contacts). The questionnaire consists of twelve items rated on a 7-point Likert scale and four yes/no items. The twelve items were summed into a total score. Cronbach’s α for this measure was good (0.84).

Percentage of surrounding greenness was measured using the Dutch Land Use database [18] and includes urban green, agricultural green, and natural green. Urban green includes vegetable gardens (>0.1 hectare (ha)), sport and recreation areas (>0.5 ha), and parks (>1 ha). The percentage of green space is calculated in circles (or “buffers”) of 1 kilometer (km) and 3 km around the 4-digit postal code centroid of the participant’s residence using a Geographical Information System (GIS). 

### 2.3. Statistical Analysis

We used two regression models in which the outcome (DASS/MANSA, respectively) was regressed on the percentage of green space. Because the DASS scores were skewed to the right, we used censored regression for the DASS model [19]. To examine moderating effects of age and gender we included interactions between green space and age, and between green space and gender. Because previous studies [9,10] suggested that age effects may be non-linear, six different age categories were used (<25; 25–34; 35–44; 45–54; 55–64; >65). Separate models were used for the 1 km and 3 km greenness buffers. The models were adjusted for several personal characteristics that may confound the association between greenness and mental health, namely: age, gender, education, partner status, employment status, origin (Dutch native/immigrant), and income. The analyses were adjusted with sampling weights using the svy command in Stata13 [20]. Weight factors were based on population proportions derived from Statistics Netherlands, derived for 36 strata based on age (six categories), gender, and education level (three categories); see [15].

## 3. Results

Descriptive characteristics of the sample are presented in Table 1. Note that DASS data were available for only 4765 participants (97%).

The regression models on the association between green space and mental health are shown in Table 2. The left two columns show the results for the 1 km green buffer, for the DASS and the MANSA, respectively. For this 1 km buffer, the interactions between green space and the different age groups were not significant, nor was the interaction between green space and gender. Without the interaction terms in the models the association between green space in a 1 km radius and the DASS score was not significant (B = −0.3, standard error (s.e.) = 1.8, *p* = 0.883). The same was true for the MANSA (B = −0.5, s.e. = 0.9, *p* = 0.569).

**Figure 1:**
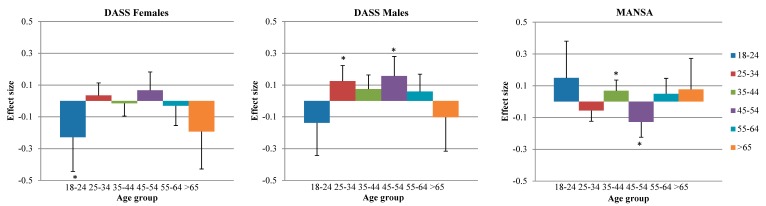
Association between percentage of green space in a 3 km radius and psychological symptoms (DASS) and quality of life (MANSA), by age group and gender. The MANSA graph is not separated by gender as the green space*gender interaction was non-significant in this model. Sample sizes for DASS: age 18–24, *n* = 278/63 (women/men); age 25–34, *n* = 568/183; age 35–44, *n* = 495/224; age 45–54, *n* = 837/372; age 55–64, *n* = 731/530; age > 65, *n* = 210/265. Sample sizes for MANSA: age 18–24, *n* = 358; age 25–34, *n* = 779; age 35–44, *n* = 739; age 45–54, *n* = 1244; age 55–64, *n* = 1307; age > 65, *n* = 497. *: effects significantly different from 0.

The right two columns of Table 2 show the results for the 3 km buffer. The models for the DASS and the MANSA showed significant interactions between green space and certain age groups, while in the DASS model also the interaction between green space and gender was significant. This suggests that the association between green space in a 3 km radius and mental health is different for people of different age groups (DASS and MANSA) and gender (DASS). Figure 1 shows the estimated effect sizes for the different age groups (DASS and MANSA) and different gender categories (DASS). With respect to the DASS, the largest effect sizes were observed for women of the youngest (18–24 years) and oldest (≥65) age group, though the latter effect was not significant (large s.e.; smaller sample size). In the other age groups, green space was not associated with psychopathology, let alone positively associated. Especially in males aged 45–54, higher percentages of green space were associated with more psychopathology. In this age group, green space was also related to lower quality of life (MANSA). Also for the MANSA, largest positive effects of green space were observed in the youngest and oldest age group, though not significantly so due to lower power. The observed effect sizes were small to moderate (max 0.23 per 1 SD green space).

## 4. Discussion

This study showed that green space in a 3 km buffer was related to lower levels of psychopathology and higher quality of life, but only in people of certain age and gender groups. The largest effect sizes were observed for women of the lowest and highest age groups. In some age groups, especially participants between 45 and 54 years old, green space was associated with worse rather than better mental health. With respect to the 1 km buffer, no significant associations between green space and mental health were found.

Our finding that especially some age and gender groups seem to benefit from green space is in line with previous observational population studies focusing on mental health [8,9]. De Vries *et al.* [8] found a stronger association between greenness and the number of (physical) symptoms in the elderly and housewives, which they explained by raising that these groups may spend more time in their living environment. This suggestion is corroborated by studies showing that green space was beneficial only for women who used this space four hours a week or more [11], and for individuals who were physically active [21]. Our finding that green space was related to worse mental health especially in people between 45 and 54 years old may also be understood in this light. Many people of this age may have the money to buy a house in a green area but lack the opportunity to make use of the green space because of a busy job and family life. In such a case, green space may become a primrose path: a pretty environment with potentially negative outcomes.

Gender differences in the response to greenness have also been found in studies focusing on somatic health [22], workplace greenery [23], and laboratory studies [24,25]. In some of these studies the impact of green was higher in males than in females [22,23,25], while other studies found no gender difference [26,27], or a stronger effect in females [24]. One explanation for these inconsistencies may be the type of outcome measure: somatic outcomes and physiological responses to green exposure [22,25] seem to be higher in males than in females, while self-report outcomes are not [24,26,27]; however, not all studies are consistent with this idea [23,24]. Of note, most of these studies did not examine gender as well as age interactions, while our study showed this may be relevant. Other explanations mentioned for gender differences in the effects of greenness are differences in usage and perception of green spaces (e.g., males are more likely than females to use parks and other green spaces for physical activity [28] and females may not use the green spaces if they perceive them as unsafe [29,30]). This is in line with the idea that the benefits of green space may largely depend on whether or not people make use of it.

Perhaps the most outstanding finding of this study is that in most cases the effect of green space was small or negligible. In the 1 km buffer no significant effects were found at all. This is in contrast with earlier nationwide studies done in the Netherlands [8,9,12,13]. In these studies a positive effect of green space on mental health was found in both 1 km and 3 km buffers. However, the sample size of these studies was very large (10,000 to 345,000). When Maas *et al.* [13] reduced their sample size to 4842 by selecting participants with data on social contacts, the association was not significant anymore. In other countries, studies with moderate sample sizes did show positive associations between greenness and health outcomes (see [3]), though not consistently so. We only found some effects of green space in the 3 km buffer, not the 1 km buffer. Larger buffers may include green spaces of larger size and better quality, such as parks and forests [31,32]. Existing evidence is equivocal in regard to which distance and which amount of greenness is relevant for mental health [3]. Positive effects are found for small areas of green space nearby (e.g., in buffers of 100–800 m), as well as for larger areas up to 3 km, though inconsistently and not always for each outcome measure [3]. 

Part of the inconsistencies in the literature may be explained by differential inclusion of covariates to adjust for confounding effects such as selective spatial mobility [3]. Different studies have used different sets of covariates. Especially the inclusion of income may have large effects, but data on income are not always available or only available at the area level. Because we adjusted our models for several variables reflecting socioeconomic status (household income, education, employment status, origin), and for age and partner status, we expect selection effects to be minor in our study. However, the inclusion of these covariates might also have removed part of the effects of green space on health. For example, extant work has shown that people of lower socioeconomic status tend to spend more time close to their homes [12], which may enhance the impact of green space on health outcomes. Further, people who find green space most important also more often decide to live in green areas [33,34]. Thus, adjusting for selective spatial mobility may have removed some of the effects of interest.

A limitation of our study is that the participants’ residences were identified with only four postal code digits, while Dutch postal codes consist of four digits and two letters. We did so because of privacy reasons. This reduced the accuracy of our greenness measure, but probably increased the reliability of our symptom measures. Lower accuracy of the greenness measure can be expected to reduce power and lead to underestimation of effects. Some effects may therefore have gone unnoticed. Future studies may also enhance accuracy by using more detailed land-use databases, enabling the differentiation of smaller patches of green spaces like street trees and gardens. In the Netherlands, a more fine-grained dataset will be available from 2017 onwards [35]. This will also enhance the possibility to study the differential effects of buffers with a smaller radius (<1 km). Other limitations of our study are the cross-sectional design and the fact that our sample was highly educated and predominantly female. We compensated for this sampling bias by using sampling weights in our analyses. A strong point is that we had person-level data on the participants’ income. Additionally, we used instruments to assess symptoms of anxiety or depression that were selected for their sensitivity in general population samples [15], and because previous work in the Netherlands suggested that these indices of mental health were most sensitive to the effects of green space [12].

## 5. Conclusions

To conclude, this study suggests that the effects of green space are small and only exist for people of specific age and gender groups. Presumably, not the presence of green space as such, but whether or not people make use of this green space determines its value.

## Figures and Tables

**Table 1:** Descriptive characteristics of the participants (*n* = 4924).

Characteristic	n (%) or Mean (s.d.)
Female, n (%)	3219 (65%)
Age group 18–24 years, n (%)	358 (7.3%)
Age group 25–34 years, n (%)	779 (15.8%)
Age group 35–44 years, n (%)	739 (15.0%)
Age group 45–54 years, n (%)	1244 (25.3%)
Age group 55–64 years, n (%)	1307 (26.5%)
Age group 65 or older, n (%)	497 (10.1%)
Level of education, mean (s.d.)	7.0 (1.1)
Having a partner, n (%)	3594 (73%)
Having paid work, n (%)	3616 (73%)
Dutch native, n (%)	4780 (97%)
Household income (euros/month), mean (s.d.)	2746 (1038)
DASS Total, median (IQR)	13 (19); range 0–126
MANSA, mean (s.d.)	62.4 (8.7); range 12–84
Green space in 1 km buffer, mean (s.d.)	29% (22%); range 0%–98%
Green space in 3 km buffer, mean (s.d.)	47% (23%); range 0%–97%

Notes: Educational level ranged from 1 (elementary school not finished) to 8 (academic degree). DASS Total = Depression Anxiety Stress scale total score (*n* = 4765); MANSA = Manchester Short Assessment of Quality of Life (*n* = 4924); IQR = Interquartile range; s.d. = standard deviation.

**Table 2:** Regression analyses for the association between green space and mental health, moderated by age and gender.

Independent Variables	1 km Buffer		3 km Buffer	
	DASS *n* = 4765	MANSA *n* = 4924	DASS *n* = 4765	MANSA *n* = 4924
Percentage of green space	−17.7 (11.3)	5.2 (5.5)	−16.9 (8.1) *	6.3 (4.5)
*Age group*				
18–24 years	ref.	ref.	ref.	ref.
25–34 years	−2.7 (3.9)	−1.2 (2.3)	−5.7 (4.5)	−0.0 (2.7)
35–44 years	−0.6 (4.0)	−4.4 (2.3) **^~^**	−4.2 (4.5)	−3.0 (2.7)
45–54 years	−2.2 (4.1)	−1.9 (2.3)	−8.0 (4.8) **^~^**	1.2 (2.8)
55–64 years	−6.5 (4.1)	−1.2 (2.3)	−8.8 (4.9) **^~^**	−0.7 (2.8)
65 or older	−9.2 (5.1)	3.5 (2.7)	−7.3 (6.8)	3.1 (3.6)
*Gender*				
Female	ref.	ref.	ref.	ref.
Male	−2.5 (1.4)	−0.6 (2.7)	−4.7 (1.8) **	−0.4 (0.9)
Green space*Age group				
Green space*18–24 years	ref.	ref.	ref.	ref.
Green space*25–34 years	22.0 (11.7) **^~^**	−9.8 (5.6) **^~^**	19.5 (8.4) *	−8.2 (4.6)^~^
Green space*35–44 years	13.9 (11.6)	−0.8 (5.6)	15.8 (8.3) **^~^**	−3.3 (4.6)
Green space*45–54 years	16.9 (11.9)	−7.1 (5.7)	21.8 (8.9) *	−10.6 (4.8) *
Green space*55–64 years	16.9 (11.9)	−4.5 (5.7)	14.6 (8.7) **^~^**	−3.8 (4.8)
Green space*65 or older	10.4 (13.1)	−5.5 (6.5)	2.6 (11.3)	−2.7 (5.9)
Green space*Gender				
Green space*Female	ref.	ref.	ref.	ref.
Green space*Male	3.3 (3.6)	−1.1 (1.8)	6.7 (3.3) *	−1.2 (1.7)

Notes: Unstandardized regression coefficients (s.e.); ref. = reference category; DASS = Depression Anxiety Stress scale total score; MANSA = Manchester Short Assessment of Quality of Life; s.e. = standard error. All models are adjusted for age, gender, level of education, partner status, employment status, origin, and household income. Percentage of green space is expressed as a fraction (0–1). **^~^** *p* < 0.10; * *p* < 0.05; ** *p* < 0.01.
